# The burden of care in hip and knee prosthetic joint infections (PJI) in a regional unit

**DOI:** 10.1007/s11845-026-04299-x

**Published:** 2026-03-26

**Authors:** Nicholas Clausen, Eoghan Pomeroy, Fiachra Rowan, Paul McKenna, May Cleary

**Affiliations:** 1 Department of Trauma and Orthopaedics, University Hospital Waterford, Waterford, Ireland; 2 Department of Trauma and Orthopaedics, University College Cork (UCC), Cork, Ireland

**Keywords:** Arthroplasty, Prosthetic Joint Infection (PJI), THA, TKA

## Abstract

**Background:**

Prosthetic joint infection (PJI) is a serious complication of joint arthroplasty. Irish patients undergo joint arthroplasty in public and private hospitals both in Ireland and abroad. Patients with complications often present to their local Emergency Department, regardless of initial point of care.

**Aims:**

We hypothesised an additional burden placed on the public system by patients with PJI that had their index surgery in an outside institution possibly due to delays in presentation.

**Methods:**

Retrospective analyses of all total hip arthroplasty (THA) and total knee arthroplasty (TKA) PJI managed by a multidisciplinary PJI team over a 3-year period (December 2021—December 2024). The means of total estimated operative costs, number of operations, length of inpatient stay (LOS), antibiotic treatment duration, admissions and rates of single operative management were compared between patients whose index surgery was done offsite and those done locally.

**Results:**

88 PJIs were managed during the study period. Mean operative cost was lower for local PJI (€49,533) compared with offsite PJI (€77,996). Local PJI patients required fewer operations (1.26 vs. 1.85) and admissions (1.16 vs. 1.48).

Among TKA-PJI, offsite cases incurred higher operative costs (€75,909 vs. €40,500), required more operations (2.27 vs. 1.23), had longer LOS (55.9 vs. 28.2 days), and more admissions (1.64 vs. 1.15). Single-operation management was achieved in 88% of local versus 45% of offsite TKA-PJI cases.

**Conclusion:**

Offsite TKA-PJI imposes a disproportionate burden on public healthcare resources and is less likely to be successfully managed with a single operation.

## Introduction

Prosthetic joint infections (PJI) are a serious complication of joint arthroplasty and care challenging for both patients and surgeons alike. Timely intervention can result in a quick resolution with reduced hospital stays and manageable antibiotic courses [1, 2]. Overall rates of PJI following total joint arthroplasty are 1–2% [3]. PJI cases are expected to rise by 150–600% by 2030 in the USA, with similar rates expected in Europe.[4]^2^ A 2017 study by Premkumar et al.[5] analysed the incidence and economic impact of PJI in the USA over a 15-year period and noted that although the growth rate of total joint arthroplasty since the late 2000 s has slowed, volume has substantially increased over the last 2 decades. This increase in total hip arthroplasty (THA) and total knee arthroplasty (TKA) case volume is a main driver for PJI with their study estimating combined annual hospital costs of PJI for hip and knee to be $1.85 billion by 2030 [5]. PJI is a leading cause of failure in primary THA, alongside instability and aseptic loosening, and rates of THA-PJI and TKA-PJI are predicted to increase by 176% and 170%, respectively, by 2030 according to recent models [6]. A recent systematic review has also shown infection to be the leading cause of failure in primary TKA [7]. It has been previously shown in the UK that revision burden for PJI is higher than that of primary joint arthroplasty[8, 9], and as rates of revision due to PJI increase so does the economic burden of the condition [10]. Furthermore, patients with THA-PJI who undergo revision cost approximately £33,000 more than THA revisions for alternate reasons [11].

A 2023 systematic review in Germany found that there is a scarcity of research data regarding PJI which is urgently needed to accurately assess the socioeconomic burden in Europe. Furthermore, a lack of data on DAIR and single-stage revision is highlighted [12]. The full economic impact of PJI treatment is extremely difficult to assess not only due to limited studies that address this issue but also due to dramatic differences in setting depending on type of healthcare system and nation-specific economic standards [13]. A recent analysis by Alt et al. extrapolated the health economic burden of PJI in 2019 and showed markedly different reimbursement payments across Europe, leading to potential disparities in access to care, further highlighting the need for healthcare policies to accurately reflect true costs associated with PJI treatment [14]. Nevertheless, septic revision is consistently around 1.5–3 times more costly than aseptic revision, and 2–4 times more expensive than index surgery [13, 15]. There is a paucity of literature on PJI prevalence and cost of inpatient care in Ireland. Kutubi et al.[16] recently published the first study in Ireland and UK regarding outpatient antibiotic therapy (OPAT) for PJI patients, in which they found a readmission rate of 29.3% and a median estimated financial benefit of €26,505 within their cohort.

With the introduction of ‘Sláintecare’, Ireland aims to provide a universal single-tier health system to level out care inequalities [17]. Currently patients can undergo their primary joint arthroplasty at public or private hospitals. Public patients have their primary arthroplasty generally in the public hospitals or, on occasion, in a private facility elsewhere funded by government initiatives to address long waiting lists (such as the National Treatment Purchase Fund (NTPF) and Cross Border Directive Scheme). Patients with private health insurance may attend a public hospital privately or attend an offsite private institution for their primary arthroplasty. In the future it is proposed that all patients with insurance have their joint arthroplasty outside of the public hospitals. However, the cost implication of future PJI management in this cohort should still be an important consideration for healthcare managers and budget planning within the public sector as these cases tend to present to the local public hospital for emergent care in the case of PJI. This study aimed to identify our incidence of hip and knee PJI presenting to our institution for care, as well as identify the referral source and examine the impact of the referral source on the overall cost of care.

## Methods

Retrospective analysis of a prospectively maintained database of THA and TKA PJI managed by a Multidisciplinary (MD) PJI team in a model 4 institution in Ireland over a 3-year period (December 2021—December 2024) was performed. Microsoft Excel (Microsoft Excel, version 16.99 for Mac, Microsoft Corporation, Redmond, Washington USA, www.microsoft.com) was used to collate the data, which included the type of intervention required (i.e., debridement, antibiotics and implant retention (DAIR) vs. single-stage revision vs. two-stage revision), length of inpatient stay, the estimated cost of the type of operative intervention and antibiotic course duration. The averages of estimated total operative costs, length of inpatient stay, and antibiotic treatment duration were then compared between patients with an index surgery at a distant site and those who had their primary arthroplasty at the local unit.

Local ethical approval was granted for the study by the Research Ethics Committee, HSE, South East (Ref 25.12). Statistical analysis was performed using Prism (GraphPad Prism, version 10.5.0 for Mac, GraphPad Software, Boston, Massachusetts USA, www.graphpad.com) using t-test and descriptive statistics to compare the study endpoints between groups (e.g. inpatient stay, total operative cost, length of antibiotic duration). For crude analysis of operative costs, means were utilised according to HSE ABF Admitted Price List 2024 [18], which served as a surrogate rather than representing true individual costings.

## Results

A total of 88 PJI were treated in the 3-year period; of these, there were 51 THA-PJI and 37 TKA-PJI. PJIs referral source were determined based on the hospital in which primary surgery occurred, including private hospitals within Ireland as well as some in the UK through the Cross Border Directive and others in Europe.

Patient demographics were recorded for each cohort, as seen in Table 1. Of all the PJI analysed in our study, 31% (27/88) had their index surgery in an offsite institution. Distribution of THA-PJI (57%; 35/61) and TKA-PJI (43%; 26/61) in the local group were similar to the THA-PJI (59%; 16/27) and TKA-PJI (41%; 11/27) in the offsite cohort. Patient age was recorded based upon date of discussion at initial MDT in which their PJI diagnosis was made. The mean age of all local PJI patients was similar to the mean age of all offsite PJI patients (*p* = 0.1250, 73.15 years vs. 70.17 years). There were also no significant differences when comparing the mean age of THA-PJI or TKA-PJI within the local and offsite cohorts (*p* = 0.2339, 72.93 years vs. 70.33 years; *p* = 0.1786, 73.43 years vs. 69.95 years, respectively). Within the local PJI cohort, 62% (38/61) of patients were male compared to 74% (20/27) of the offsite PJI cohort. The time interval between index surgery and presentation to our unit was recorded where possible, however 2 offsite (2 THA-PJI) and 10 local (6 THA-PJI and 4 TKA-PJI) patients were excluded from calculations as the data was unavailable. The mean time interval since index surgery for offsite PJI patients was 7.00 years (range: 0–25) compared to 5.92 years (*p* = 0.3038; range: 0–30) in the local PJI cohort. Offsite THA-PJI patients presented a mean of 9.00 years (range: 0–25) after index surgery compared to a mean of 5.76 years (*p* = 0.1434; range: 0–30) in the local THA-PJI cohort. The offsite TKA-PJI cohort presented a mean of 4.455 years (range: 0–13) since index surgery compared to 6.14 years (*p* = 0.2775; range: 0–30) in the local TKA-PJI cohort.

**Table 1:** Patient demographics

	**Local**	**Offsite**	
	**Mean ± SD**	**Mean ± SD**	
**# Total PJI**	61	27	
**# THA-PJI**	35	16	
**# TKA-PJI**	26	11	
**M:F Total**	38:23	20:7	
**M:F THA-PJI**	25:10	12:4	
**M:F TKA-PJI**	13:13	8:3	
**Age Total PJI (yrs)**	73.15 ± 11.54	70.17 ± 10.08	*p* = 0.1250
**Age THA-PJI (yrs)**	72.93 ± 12.58	70.33 ± 9.88	*p* = 0.2339
**Age TKA-PJI (yrs)**	73.43 ± 10.20	69.95 ± 10.85	*p* = 0.1786
**Interval Since Index Op (Total) (yrs)**	5.92 ± 8.83	7.00 ± 7.98	*p* = 0.3038
**Interval Since Index Op (THA-PJI) (yrs)**	5.76 ± 9.23	9.00 ± 9.23	*p* = 0.1434
**Interval Since Index Op (TKA-PJI) (yrs)**	6.14 ± 8.48	4.46 ± 5.43	*p* = 0.2775

PJI = prosthetic joint infection; THA-PJI = total hip arthroplasty prosthetic joint infection; TKA-PJI = total knee arthroplasty prosthetic joint infection.

Characteristics of inpatient management for each cohort was assessed, as seen in Table 2. There was a significant difference (*p* = 0.0027) in the average estimated cost of operative treatment for patients with PJI whose index THA or TKA was performed at an offsite institution (mean €77,996; range: €24,700 – €244,000) compared to patients whose index procedure was performed locally (mean €49,533; range: €0 – €185,700). The estimated operative cost of local TKA-PJI (mean €40,500; range: €0—€120,600) was significantly different (*p* = 0.0026) to offsite TKA-PJI (mean €75,909; €26,400 – €172,200). There was a nonsignificant difference (*p* = 0.0624) between estimated operative costs of local THA-PJI (mean €56,243; range: €0—€185,700) and offsite THA-PJI (mean €79,431; range: €24,700—€244,000).

**Table 2:** Inpatient Management

	**Local**	**Offsite**	
	**Mean ± SD**	**Mean ± SD**	
**Op Cost (Total) (€)**	49,533 ± 36,651	77,996 ± 55,464	**p = 0.0027
**Op Cost (THA-PJI) (€)**	56,243 ± 43,677	79,431 ± 59,866	*p* = 0.0624
**Op Cost (TKA-PJI) (€)**	40,500 ± 21,947	75,909 ± 51,128	**p = 0.0026
**# Ops (Total)**	1.26 ± 0.63	1.85 ± 1.26	**p = 0.0022
**# Ops (THA-PJI)**	1.29 ± 0.71	1.56 ± 0.89	*p* = 0.1198
**# Ops (TKA-PJI)**	1.23 ± 0.51	2.27 ± 1.62	**p = 0.0025
**LOS (Total) (days)**	43.49 ± 46.38	46.11 ± 49.06	*p* = 0.4054
**LOS (THA-PJI) (days)**	54.86 ± 54.37	39.38 ± 39.95	*p* = 0.1568
**LOS (TKA-PJI) (days)**	28.19 ± 26.80	55.91 ± 60.72	*p = 0.0298
**ABX (Total) (weeks)**	16.04 ± 12.56	13.86 ± 5.85	*p* = 0.2204
**ABX (THA-PJI) (weeks)**	16.37 ± 14.79	12.00 ± 5.27	*p* = 0.1637
**ABX (TKA-PJI) (weeks)**	15.59 ± 8.97	16.10 ± 5.97	*p* = 0.4358
**PO ABX (On D/C) (Total) (weeks)**	5.83 ± 5.45	6.09 ± 5.96	*p* = 0.4250
**PO ABX (On D/C) (THA-PJI) (weeks)**	4.29 ± 3.84	6.30 ± 4.52	*p* = 0.0664
**PO ABX (On D/C) (TKA-PJI) (weeks)**	8.09 ± 6.67	5.78 ± 7.90	*p* = 0.2048
**# Admissions (Total)**	1.16 ± 0.45	1.48 ± 0.70	**p = 0.0064
**# Admissions (THA-PJI)**	1.17 ± 0.45	1.38 ± 0.72	*p* = 0.1122
**# Admissions (TKA-PJI)**	1.15 ± 0.46	1.64 ± 0.67	**p = 0.0083

PJI = prosthetic joint infection; THA-PJI = total hip arthroplasty prosthetic joint infection; TKA-PJI = total knee arthroplasty prosthetic joint infection; LOS = length of stay; ABX = antibiotics; PO ABX = oral antibiotics; D/C = discharge.

Local PJI patients underwent fewer operations on average (mean 1.26; range 0–4) compared to PJI patients from an offsite institution (mean 1.85; range 1–6), which was significant (*p* = 0.0022). There was also a significant difference (*p* = 0.0025) in the average number of operations when comparing local TKA-PJI (mean 1.23; range 1–3) to offsite TKA-PJI (mean 2.27; range: 1–6). The average number of operations for THA-PJI in the local (mean 1.29; range 0–4) and offsite (mean 1.56; range 1–4) cohorts was similar.

The total number of inpatient days each patient spent in hospital receiving treatment for their specific PJI was recorded. PJI patients referred from elsewhere stayed an average of 46.11 days (range: 6–225) in hospital compared to 43.49 days (*p* = 0.4054; range: 7–216) in the local PJI cohort. Local THA-PJI patients stayed an average of 54.86 days (range: 7–216) compared to 39.38 days (*p* = 0.1568; range: 6–146) in the offsite cohort. There was a significant difference in the average length of hospital stay (*p* = 0.0298) between the local TKA-PJI patients (mean 28.19 days; range: 8–115) and offsite TKA-PJI patients (mean 55.91 days; range: 11–225).

PJI patients from outside units had an average antibiotics course of 13.90 weeks (range: 4–26) on discharge compared to 16.04 weeks (*p* = 0.2300; range: 0–76) for the local PJI patients. The average antibiotic course length in the offsite THA-PJI (mean 12.00 weeks; range: 4–24) and local THA-PJI (mean 16.37 weeks; range: 0–76) cohorts was not significantly different (*p* = 0.1637). There was no significant difference (*p* = 0.3984) between the antibiotic course length of local TKA-PJI (mean 15.59 weeks; range: 0–36) and offsite TKA-PJI (mean 16.44 weeks; range: 12–26). Of note, some patients from the offsite PJI group (6 Total; 4 THA, 2 TKA) and local PJI (9 Total; 5 THA, 4 TKA) cohorts were excluded from calculations in both groups due to indefinite end-of-treatment dates or death during their inpatient stay. Regarding these patients that were excluded, 4 THA-PJI (25.00% of total) and 2 TKA-PJI (18.18% of total) patients of the offsite cohort required indefinite antibiotic courses compared to 0 THA-PJI (0% of total) and 2 TKA-PJI (7.69% of total) patients in the local cohort. There were no deaths in the offsite PJI cohort while there were 5 THA-PJI (14.29% of total) and 2 TKA-PJI (7.69% of total) patients in the local PJI cohort who died while as inpatients.

Regarding intravenous to oral switch of antibiotics on final discharge, offsite PJI patients had an average oral antibiotic course of 6.09 weeks (range: 0–20) compared to an average of 6.39 weeks (*p* = 0.4198; range: 0–24) in the local PJI group. The average oral antibiotic course length in the offsite THA-PJI (mean 6.33 weeks; range: 0–12) and local THA-PJI (mean 5.03 weeks; range: 0–18) cohorts was not significantly different (*p* = 0.1749). There was no significant difference (*p* = 0.2006) between the oral antibiotic course length of local TKA-PJI (mean 8.18 weeks; range: 0–24) and offsite TKA-PJI (mean 5.78 weeks; range: 0–20). Some patients from the offsite PJI group (6 Total; 4 THA, 2 TKA) and local PJI (9 Total; 5 THA, 4 TKA) cohorts were excluded from calculations in both groups due to indefinite end-of-treatment dates or death during their inpatient stay.

The number of admissions that took place during the course of treatment for each PJI patient was further analysed. The local PJI cohort had fewer admissions on average (mean 1.16 admissions; range 1–3) compared to the PJI cohort from an outside institution (mean 1.48 admissions; range 1–3), which was statistically significant (*p* = 0.0064). When analysing the THA-PJI and TKA-PJI subgroups between the cohorts, the average number of admissions was similar for THA-PJI, however the local TKA-PJI group had fewer admissions (mean 1.15 admissions; range: 1–3) than the offsite TKA-PJI group (mean 1.64; range 1–3), which was also significant (*p* = 0.0083).

The rates of patients requiring single operative management was analysed, including rates of DAIR vs. non-DAIR, as seen in Table 3. In the PJI cohort referred from elsewhere, 56% (15/27) had a single operation. Of these, 33% (9/27) of the offsite PJI cohort underwent DAIR procedures as their only operation: 31% (5/16) of THA-PJI and 36% (4/11) of TKA-PJI. The remaining 22% (6/27) underwent first-stage revision as their only operation: 31% (5/16) of THA-PJI and 9% (1/11) of TKA-PJI.

**Table 3:** Rates of single operative management

	**Local**	**Offsite**
	**N (%)**	**N (%)**
**Single Operation (Total)**	44/61 (72%)	15/27 (56%)
**Single Operation (THA-PJI)**	24/35 (69%)	10/16 (63%)
**Single Operation (TKA-PJI)**	20/26 (88%)	5/11 (45%)
**DAIR PJI (Total)**	20/61 (33%)	9/27 (33%)
**DAIR PJI (THA-PJI)**	11/35 (31%)	5/16 (31%)
**DAIR PJI (TKA-PJI)**	9/26 (35%)	4/11 (36%)
**First-Stage Revision (Total)**	21/61 (34%)	6/27 (22%)
**First-Stage Revision (THA-PJI)**	10/35 (29%)	5/16 (31%)
**First-Stage Revision (TKA-PJI)**	11/26 (42%)	1/11 (9%)
**Girdlestone (THA-PJI)**	3/35 (9%)	0/16 (0%)
**Non-Op (Total)**	4/61 (7%)	0/27 (0%)
**Non-Op (THA-PJI)**	3/35 (9%)	0/16 (0%)
**Non-Op (TKA-PJI)**	1/25 (4%)	0/11 (0%)

PJI = prosthetic joint infection; THA-PJI = total hip arthroplasty prosthetic joint infection; TKA-PJI = total knee arthroplasty prosthetic joint infection; DAIR = debridement, antibiotics, and implant retention.

In the local PJI cohort, 72% (44/61) of patients had a single surgery. 33% (20/44) of local THA-PJI and TKA-PJI underwent DAIR procedures as their only operation: 31% (11/35) of THA-PJI and 35% (9/26) TKA-PJI. A total of 34% (21/61) local PJI patients underwent a first-stage revision as their only operation: 29% (10/35) of THA-PJI and 42% (11/26) of TKA. Of the remaining seven patients in the local PJI cohort, four patients (3 THA-PJI and 1 TKA-PJI) underwent non-operative management and 3 THA-PJI underwent girdlestone procedures.

## Discussion

Our analysis of PJI patients over a three-year period shows an apparent discrepancy in the economic burden of PJI patients who underwent their index surgery in an offsite institution (such as private hospital, National Treatment Purchase Fund (NTPF) or Cross Border Directive Scheme) compared to local PJI patients. The offsite PJI cohort had greater mean total operative costs, number of operations, and number of admissions (€77,996; 1.852; 1.481, respectively) compared to the local PJI cohort (€49,533; 1.262; 1.164, respectively). Most notably, the TKA-PJI subgroup within the offsite cohort had these same trends and additionally had longer mean LOS (46.11 days) than TKA-PJI from the local PJI cohort (28.19 days). Furthermore, when comparing rates of single operative management between both cohorts, rates of DAIR and first-stage revision were similar across the subgroups, except in single-stage revision for TKA-PJI, where there was over a 30% difference in the local (42%) and offsite (9%) cohorts.

Criteria for DAIR is standardised in our institution and discussed at our weekly PJI MDT, which likely played a significant role in the proportional similarities between each cohort’s subgroups. However, it is not clear what caused the discrepancy within the single stage revision rates. One possibility is that patients in the offsite cohort had procedures remote from their home, thus their postoperative care may default to their general practitioner or local community hospital. With either scenario, they may have incurred a delay in presentation to our unit or were partially treated with antibiotics leading to no identifiable pathogen on aspiration, which may preclude single-stage revision. There were no significant differences in time interval since index operation across the two cohorts and their subgroups. However, because these patients received care in several hospitals or countries before presenting to our unit, it is difficult to compile detailed information regarding treatment they may have received in the interim before presenting to our unit.

Single-stage revisions for THA-PJI have been proven to be advantageous regarding reduced overall cost and operative time compared to multi-stage revisions due to the inherent nature of explanation and reimplantation within the same procedure [19]. Appropriate patient selection for DAIR and single-stage revision can have similar success rates to two-stage revision, but with lower morbidity, better function and lower hospital costs [20]. Single-stage revisions have been shown to have lower intraoperative complications and better patient-reported outcomes at 3 months when compared to two-stage revisions [21]. Evidence shows that single-stage exchange for TKA-PJI with strict patient selection criteria can lead to a re-infection rate of 0% at three year follow-up [22]. Furthermore, single-stage revisions for TKA and THA PJI have been shown to have outcomes in elderly patients equal to that in younger cohorts [23]. In our institution, we have a strict criteria for single-stage revision, as described in the above referenced study, thus it is possible that the low rate of single-stage revision in the offsite TKA-PJI cohort can be attributable to culture-negative PJI from antibiotic exposure prior to admission to our unit.

Regarding total estimated operative costs between both cohorts, the greater economic burden experienced in the offsite PJI group is most notably impacted by the TKA-PJI subgroup. Unlike revision for THA-PJI, infection has been shown to be the most common aetiology for revision TKA, placing significant socioeconomic burden on the US health system with a mean cost of $75,028 [24]. Our method of estimating operative cost based on activity-based funding (ABF) and Diagnosis Related Groups (DRG) coding was previously done in a recent Australian study by Hammat et al. in which they found the mean total hospital cost per PJI patient was $64,585 Australian dollars (AUD), or an excess of $250 million (AUD) to the Australian healthcare system [25]. Similar to the data we’ve analysed, they noted a difference in mean LOS of TKA-PJI when patients underwent single-stage revision (mean 35.7 days) compared to two-stage revision (mean 59.4 days) [25].

Surgery remains the gold standard treatment in PJI; long-term antibiotic courses alone will not eradicate the infection and raise concerns of resistance development and are not well tolerated well by patients [20]. When PJI is suspected or confirmed, well-defined care pathways and MDT involving infectious disease physicians and microbiologists is vital to reduce the likelihood of re-infection[26], as outlined in British Orthopaedic Association Standards for Trauma and Orthopaedics (BOAST) guidelines [20, 27]. For example, a recent study analysed the PJI microbiome in Ireland, finding prevalence rates of coagulase negative staphylococci (CoNS) and staphylococcus aureus (SA) similar to previous studies done in the US, leading to empiric anti-microbial guideline implementation in coordination with their microbiology and infectious disease colleagues [28]. With carefully selected patients and structured criteria, single-stage revision could lead to greater rates of infection eradication while also reducing costs associated with additional operations, theatre time and hospital bed-days.

The management of patients with complex musculoskeletal infection by multi-disciplinary teams including orthopaedic surgeons, infectious disease physicians, microbiologists, intensivists and plastic surgeons has been shown to reduce re-infection rates and patient mortality [29]. When compared to aseptic revisions, PJI revisions have significantly higher risk of postoperative complications including sepsis and nonhome discharge, but also readmission, prolonged hospital stay, re-revision and death, similar to our analysis of this study’s cohort [30, 31]. Complication rates during interstage and post-reimplantation stages are high, with one study finding 27% of patients experiencing medical and surgical complications.[32]With the introduction of Slaintecare, the future proposition that all patients with insurance have their primary joint arthroplasty outside of public hospitals will likely increase the rates of offsite PJI patient presentation. Prevalence of primary THA and TKA are expected to double by 2030[5] and volume of PJI cases are expected to increase over the same period [6]. If these expected rates are further exacerbated by presentation of offsite PJI patients, that we have shown in our study to require a greater inpatient management, there were will be an additional economic burden that cannot be ignored. While we believe that these complex infections should be managed in institutions with the expertise and MDT input to achieve the best outcomes, we also believe that healthcare systems need to account for the burden that these patients place on services within the tertiary referral units.

There are a few limitations with our study. It is difficult to assess the entire economic burden of these PJI patients as we did not factor in costs associated with OPAT or specific antibiotic regimen, follow-up clinics, or operations outside the study period, rather we analysed cost using surrogate data as described. Furthermore, as with any retrospective study based on an MDT database, it is possible that patients may have been inadvertently excluded from the study if they were not discussed at the MDT. There were also issues with inconsistent data reporting which were uncovered during the data collection period which led to an internal audit and creation of a standardized MDT template to prevent these issues in the future. This is evident in the lack of detailed information regarding treatment these patients may have received in the interim before presenting to our unit. Additionally, MDT notes were not regularly updated regarding which patients were culture-negative until later in the data collection period, thus making it hard to accurately analyze rates of culture-negative PJI in these patient cohorts.

## Conclusion

Our data confirms the high cost of PJI care in our institution with an average of €49,533 for local PJI and €77,996 for offsite PJI, consistent with previous literature. Additionally, patients in the offsite PJI cohort had greater number of operations and number of admissions compared to the local PJI cohort (p-value = 0.0022 and p-value = 0.0064, respectively).

TKA-PJI patients whose index procedure was performed at another institution have a statistically significant increased total operative cost, number of operations, LOS and number of admissions when compared to the local cohort (p-value = 0.0027, p-value = 0.0025, *p* = 0.0298 and p-value = 0.0083, respectively). Furthermore, 88% of local TKA-PJI patients were effectively managed with a single operation compared to 45% of offsite TKA-PJI patients.

PJI care confers a significantly higher operative cost on the public health system when the index surgery is performed in an external institution. This has important consequences for healthcare planning in the context of the implementation of Sláintecare, with proposed cessation of private arthroplasty operations in public hospitals. This data demonstrates that this could lead to a disproportionate increase in the cost of PJI management within the public system as a result. Healthcare resource planning for increased healthcare costs in publicly funded hospitals for treatment of PJI should be an important consideration in the future.
